# Interpretation of machine learning models using shapley values: application to compound potency and multi-target activity predictions

**DOI:** 10.1007/s10822-020-00314-0

**Published:** 2020-05-02

**Authors:** Raquel Rodríguez-Pérez, Jürgen Bajorath

**Affiliations:** Department of Life Science Informatics, B-IT, LIMES Program Unit Chemical Biology and Medicinal Chemistry, Rheinische Friedrich-Wilhelms-Universität, Endenicher Allee 19c, 53115 Bonn, Germany

**Keywords:** Machine learning, Black box character, Structure–activity relationships, Compound activity, Compound potency prediction, Multi-target modeling, Model interpretation, Feature importance, Shapley values

## Abstract

Difficulties in interpreting machine learning (ML) models and their predictions limit the practical applicability of and confidence in ML in pharmaceutical research. There is a need for agnostic approaches aiding in the interpretation of ML models regardless of their complexity that is also applicable to deep neural network (DNN) architectures and model ensembles. To these ends, the SHapley Additive exPlanations (SHAP) methodology has recently been introduced. The SHAP approach enables the identification and prioritization of features that determine compound classification and activity prediction using any ML model. Herein, we further extend the evaluation of the SHAP methodology by investigating a variant for exact calculation of Shapley values for decision tree methods and systematically compare this variant in compound activity and potency value predictions with the model-independent SHAP method. Moreover, new applications of the SHAP analysis approach are presented including interpretation of DNN models for the generation of multi-target activity profiles and ensemble regression models for potency prediction.

## Introduction

Major tasks for machine learning (ML) in chemoinformatics and medicinal chemistry include predicting new bioactive small molecules or the potency of active compounds [1–4]. Typically, such predictions are carried out on the basis of molecular structure, more specifically, using computational descriptors calculated from molecular graph representations or conformations. For activity prediction, ML models are trained to systematically associate structural patterns, represented in more or less abstract forms, with known biological activities of small molecules. Classification models are derived for predicting class labels of test compounds (e.g., active/inactive or highly/weakly potent) whereas regression models predict numerical potency values. Supervised ML can also be applied to predict other molecular properties.

Understanding model decisions is generally relevant for assessing the consistency of predictions and detecting potential sources of model bias. Interpretability is also crucial for extracting knowledge from modeling efforts. Accordingly, there is high interest in better understanding the basis of correct ML predictions or failures [5–9]. For example, in structure–activity relationship (SAR) analysis, explainable model decisions help to identify chemical changes that correlate with dependent variables and result in defined activity states or potency values. Having access to such model-intrinsic information enables knowledge-based validation of models and hypothesis generation [9]. In addition to model accuracy, interpretability of predictions is a major criterion for the acceptance of computational approaches in pharmaceutical research.

A shortcoming of many ML approaches is the difficulty to rationalize predictions. Lack of interpretability might result from intrinsic black box character of ML methods such as, for example, neural network (NN) [10] or support vector machine (SVM) [11] algorithms. Furthermore, it might also result from using principally interpretable models such a decision trees (DTs) as large ensembles classifiers such as random forest (RF) [12]. For a given method, lack of interpretability applies regardless of whether it is used for object classification or as an algorithmic variant for the prediction of numerical values. For example, while SVM is applied for classification support vector regression (SVR) is used for value prediction [13] and both variants yield models with black box character.

Interpretation of ML predictions can be attempted in a model-specific or model-independent (agnostic) manner. For example, feature weighting is a model-specific approach to identify descriptor contributions that determine predictions of ML models [7, 14]. However, while feature weighting is straightforward to apply to simple models, such models typically have limited predictive performance and thus also limited relevance and need for interpretation.

Notably, model interpretation can be globally attempted or at the level of individual predictions. Feature weighting approaches typically rely on a global assessment of weights or importance values for a given model and training data set. On the other hand, model decisions can also be explained focusing on individual predictions and their feature contributions. By analyzing multiple predictions, general feature trends might be detected.

As a model-independent approach, sensitivity analysis can be used to investigate the influence of systematic feature value alterations on model performance [15]. Sensitivity analysis has been applied to ML models including NNs [16] but becomes quickly computationally infeasible with increasing model dimensionality [9]. For practical purposes, sensitivity analysis is only applicable as a local adaptation by applying perturbations to individual features and examining their influence on model performance [9, 17]. Model-specific approaches require finding an appropriate compromise between model performance and interpretability, taking performance criteria for individual models into account [18]. By contrast, this is not the case for model-independent approaches, hence providing a substantial advantage. However, currently there is no agnostic approach for ML model interpretation available that would be generally applicable and serve as a standard.

Recently, we have introduced a new methodology for ML model interpretation in chemoinformatics and medicinal chemistry, which is generally applicable to ML approaches of any complexity [19]. The Shapley Additive exPlanations (SHAP) method [19, 20] is based upon the Shapley value concept [20, 21] from game theory [22, 23] and can be rationalized as an extension of the Local Interpretable Model-agnostic Explanations (LIME) approach [8]. Herein, we further evaluate the SHAP methodology by comparing local approximations and exact Shapley value calculations and report novel applications including the interpretation of potency value predictions and multi-target modeling.

## Materials and methods

### Compounds and activity data

For model building and interpretation, different compound data sets were investigated. Binary classification and regression models were generated for 10 activity classes reported in Table 1. In addition to activity classes, a large set of kinase inhibitors was assembled, as further detailed below.

**Table 1:** Compound activity classes

CHEMBL ID	Target	# Compounds	Mean pK_i_	IQR pK_i_
245	Muscarinic acetylcholine receptor M3	646	7.9	2.4
4860	Apoptosis regulator Bcl-2	620	8.5	3.3
231	Histamine H1 receptor	607	7.2	1.7
223	Alpha-1d adrenergic receptor	467	7.5	1.7
1889	Vasopressin V1a receptor	426	7.2	1.8
3798	Calcitonin gene-related peptide type 1 receptor	414	7.8	2.3
3473	C–C chemokine receptor type 3	386	7.1	1.5
4616	Ghrelin receptor	386	7.2	1.0
287	Sigma opioid receptor	345	7.4	1.3
268	Cathepsin K	331	6.9	2.0

For 10 activity classes, the CHEMBL identifier (ID), target name, number of compounds, and the average and interquartile range (IQR) of the pK_i_ value distribution are reported

Compounds and activity data were extracted from the ChEMBL database [24] and filtered as follows. Only compounds tested in target-based direct binding assays with a maximum ChEMBL assay confidence score of 9 were considered. For binary activity prediction, classification models were used to distinguish between active and inactive compounds. Therefore, inactive compounds were required as training and test instances. Assumed inactives were randomly selected from the ZINC database [25]. For training and testing of classification models, random samples of 1000 compounds each were drawn from ZINC.

#### Activity classes

For activity classes used for potency value prediction, only equilibrium constants (pKi values) were considered as potency measurements. For each compound, the mean of all available pK_i_ values was calculated, provided these values fell within the same order of magnitude (otherwise, the compound was omitted). For compounds with single or multiple potency measurements, a final pK_i_ value of at least 5 was required to exclude very weakly potent compounds from modeling. The selected activity classes contained at between 331 and 646 compounds (Table 1), which was considered a reasonable size for model building and evaluation.

#### Kinase inhibitors

The kinase inhibitor data set used herein was assembled previously [26]. To obtain a large number of kinase inhibitors, in this case, IC_50_ values were selected as potency measurements. In total, the data set contained 19,030 inhibitors of 103 human kinases.

For our analysis, these inhibitors were divided into target-based highly potent (pIC_50_ ≥ 8) and weakly potent (pIC_50_ ≤ 6) inhibitors (compounds with intermediate potency values were omitted) to control the potential influence of boundary effects on predictions. The inhibitors formed 11,120 highly potent and 11,252 weakly potent compound-kinase interactions, with an activity annotation density of ~ 1.1% of all theoretically possible inhibitor-kinase interactions. On the basis of the applied potency criteria, the final data set contained 739 multi-kinase inhibitors.

### Molecular representation

Model interpretation inherently depends on the interpretability of the descriptors or features that are used to represent compounds. Herein, the extended-connectivity fingerprint of diameter 4 (ECFP4) was used as molecular representation [27]. ECFP4 encodes layered atom environments using integers produced by a hashing function. From each compound-dependent feature set, a folded version with a constant size of 1024 bits was obtained by modulo mapping. Folded ECFP4 encodes the presence (bit set on) or absence (bit set off) of layered atom environments accounting for molecular topology.

During ECFP4 generation, the correspondence between structural patterns and bit indices was recorded and stored for visualization. For each compound, atom environments were stored as SMARTS patterns for further analysis and visualization. This made it possible to map selected ECFP4 features back onto compounds. Fingerprint calculations were implemented using Python scripts based on the *OEChem toolkit* [28].

### Model building and validation protocol

For all compound sets, data splitting was based upon computationally determined analog series [29]. Accordingly, an identified analog series was either added to the training or test set, thereby ensuring that training and test sets did not contain structural analogs from given series (which might facilitate “easy” predictions). For activity classes, compounds were divided into 70% training and 30% test data; for kinase inhibitors, 75% training and 25% test data were used. For classification models, random samples of 1000 compounds each were drawn from ZINC for training and testing, as stated above.

Cross-validation was performed using training data to select best hyperparameters for each ML model, as further specified below for each algorithm. Once hyperparameters were determined, a final model was trained for test set predictions.

Model performance was estimated on the external test set using multiple metrics. For classification results, area under the ROC curve (AUC), Matthew’s correlation coefficient (MCC) [30], and balanced accuracy (BA) [31] were calculated. MCC and BA are defined on the basis of true positive (TP), true negative (TN), false positive (FP), and false negative (FN) instances.

$$MCC = \frac{{TP \times TN - FP \times FN}}{{\sqrt {\left( {TP + FP} \right)\left({TP + FN} \right)\left({TN + FP} \right)\left( {TN + FN} \right)} }}$$

$$BA = \frac{1}{2}\left( {\frac{TP}{{TP + FN}} + \frac{TN}{{TN + FP}}} \right)$$

To evaluate regression models, the mean absolute error (MAE), mean squared error (MSE), and coefficient of determination (R^2^) were calculated. MAE, MSE, and R^2^ are defined below by where $n$ is the number of compounds, ${y}_{i}$ and ${\widehat{y}}_{i}$ are the measured and predicted pK_i_ values for compound *i*, respectively, and  is the mean.

$$MAE = \frac{1}{n}\mathop \sum \limits_{i = 1}^{n}\mid y_{i} - \hat{y}_{i} {\text{}}\mid$$

$$MSE = \frac{1}{n}\mathop \sum \limits_{i = 1}^{n} \left( {y{|}i - \hat{y}_{i} } \right)^{2}$$

### Machine learning algorithms

#### Decision trees

A decision tree (DT) is a supervised ML method that infers a sequence of binary decision rules. DT can be applied to classification and regression problems. Starting from a root node, the DT structure divides training data into subsets to optimize class label separation. DT is recursive partitioning algorithm, which iteratively generates child nodes that might be further divided into node pairs. Since the decision path from the root to the terminal or leaf nodes records features selected for predictions, DT represents an interpretable ML method. However, DTs are frequently prone to overfitting and hardly ever used as individual models for practical applications. Instead, they are typically combined to yield ensemble classifiers. In-house Python scrips based on scikit-learn [32] were used to generate all DT-based models.

#### Random forest

RF is one of the most popular ensembles of DTs [12]. Generation of the RF ensemble is based upon bootstrap aggregating and feature bagging to reduce the variance of individual trees. These approaches consider distinct compound subsets for training different DTs and random feature subsets for node splitting. Consensus predictions across all DTs forming an RF are determined and for RF regression, the average of predicted values is taken.

Herein, the number of DTs per RF was set to 300 and three hyper parameters were optimized via internal cross-validation including the maximum number of features considered at each split point (square root, log_2_) and the minimum number of samples required per internal (2, 8, 16) and leaf (1, 5, 10) nodes. For other hyperparameters, default values from scikit-learn [32] were used.

#### Extremely randomized trees

The extremely randomized trees (ExtraTrees) method is algorithmically related to RF and also based on a DT ensemble [33]. In this ensemble variant, the algorithm fully randomizes the choice of features and their values for node splitting. Moreover, ExtraTrees does not use a bootstrap sample but the entire compound training set. The main motivation behind this algorithmic variant is further reducing DT-based variance. Hyperparameter optimization corresponded to RF.

#### Gradient boosting

The gradient boosting (GB) method builds sequential DT models focusing on the errors of the previous trees [34, 35]. The prediction of each new DT aims to further improve ensemble performance. Thus, at each step, a DT is added to the GB model to minimize prediction errors via gradient descent. Here, GB regression models were built using average accuracy as a first approximation and subsequently fitting individual DTs to the model pseudo-residuals using least squares. The learning rate weights the prediction of the residuals of each individual DT and represents a hyperparameter. It was optimized via internal cross-validation (with candidate values of 0.001, 0.01, 0.1, 0.2). Other optimized hyperparameters included the maximum depth of the trees (4, 6, 8, 10), the minimum number of samples required for a leaf node (1, 5) and for sub-diving an internal node (2, 8), and the consideration of stochastic GB (with candidate values for the subsampling fraction of 1.0, 0.75, and 0.25) [35].

#### Feedforward deep neural networks

A deep neural network (DNN) consists of a series of connected units organized in sequential layers [10, 36, 37]. The basic DNN architecture includes an input layer, multiple hidden layers, and an output layer. The units are the neurons (basis functions). Neurons within the same layer act in parallel and transform input values received from the previous layer into a scalar value. Gradient descent is used to minimize the loss and backpropagation [37] to calculate the gradient of the cost function. For multi-target activity prediction, multi-task DNNs (MT-DNNs) with multiple output neurons were generated. The number of hidden layers and neurons per layer were selected across different architectures via cross-validation (with options [200, 100], [2000, 1000], and [2000, 1000, 100]). The learning rate was optimized with candidate values of 0.01 and 0.001. The batch size and dropout rate were set to 256 and 25%, respectively. Finally, the rectified linear unit (ReLU) was selected as the activation function and the number of epochs was set to 500. For internal validation, the best model was retained. DNN models were implemented with TensorFlow [38] and Keras [39].

### Principles of the SHAP methodology

#### Shapley values

The Shapley value (SHAP) concept was originally developed to estimate the importance of an individual player in a collaborative team [20, 21]. This concept aimed to distribute the total gain or payoff among players, depending on the relative importance of their contributions to the final outcome of a game. Shapley values provide a solution to the assignment of a fair or reasonable reward to each player and represent a unique result characterized by the following natural properties or axioms: local accuracy (additivity), consistency (symmetry), and nonexistence (null effect) [21].

In the context of activity predictions, Shapley values can also be rationalized as a fair or reasonable allocation of feature importance given a particular model output [19]. Features contribute to the model’s output or prediction with different magnitude and sign, which is accounted for by Shapley values. Accordingly, Shapley values represent estimates of feature importance (magnitude of the contribution) as well as the direction (sign). Features with positive sign contribute to the prediction of activity, whereas features with negative sign contribute to the prediction of inactivity (i.e., negative contribution to activity prediction).

In particular, the importance of a feature *i* is defined by the Shapley value in Eq. 1:

1 $$\phi_{i} = \frac{1}{\left| N \right|!}\mathop \sum \limits_{{S \subseteq N\backslash \left\{ i \right\}}} \left| S \right|!\left( {\left| N \right| - \left| S \right| - 1} \right)!\left[ {f\left( {S \cup \left\{ i \right\}} \right) - f\left( S \right)} \right]$$

Here $f\left(S\right)$ corresponds to the output of the ML model to be explained using a set $S$ of features, and $N$ is the complete set of all features. The final contribution or Shapley value of feature *i* (${\phi }_{i}$) is determined as the average of its contributions across all possible permutations of a feature set. Accordingly, features are individually added to the set and the change in model output reveals their relevance. Importantly, this formalism considers feature orderings, which influence the observed changes in a model’s output in the presence of correlated features.

#### Local explanations

Interpretable ML models enable rationalization of their decisions. Thus, understanding the reasons why a prediction is made by a complex model reduces or eliminates its black box character. For the explanation of individual predictions, a global understanding of the ML model is not essential. Instead, local approximations or explanations are sufficient to rationalize model decisions. Explanations of individual decisions were proposed by Ribeiro et al. and designated as Local Interpretable Model-agnostic Explanations (LIME) [8]. The LIME approach aims to find a simple model that locally approximates a complex ML model in the vicinity of a given test instance or prediction that should be explained. In this case, the test instance is an active or inactive compound. Such local explanatory models might be defined as a linear function of binary variables following Eq. 2:

2 $$g\left( {x^{\prime}} \right) = \phi_{0} + \mathop \sum \limits_{i = 1}^{\left| N \right|} \phi_{i} x^{\prime}_{i}$$

where $x^{\prime} \in \{ 0,1\} ^{{\left| {\text{N}} \right|}}$, and ${\phi }_{i}\in R$ [8]. Thus, a suitable local explanatory model is obtained by minimizing a loss function and penalizing model complexity through a regularization term according to Eq. 3:

3 $$\xi \left( x \right) = \mathop {{\text{argmin}}}\limits_{g} {\mathcal{L}}\left( {f,g,\pi_{x} } \right) + {\Omega }\left( g \right)$$

Here $f$ is the original ML model, ${\pi }_{x}$ is a kernel function, and $\Omega$ the regularization term.^8^ The kernel function defines similarity with respect to the instance $x$ to explain and therefore determines model locality.

These feature attributions from LIME might be expressed as Shapley values, which provide a LIME solution meeting the axioms listed above. Given the computational costs associated with determining exactly Shapley values according to Eq. 1, a model-independent approximation can be considered [19, 20].

#### Model-independent SHAP: kernel function

The model-independent SHAP approach or kernel SHAP is based upon an extension of LIME. Specifically, the parameters in Eq. 3 (i.e., loss, kernel, and complexity) are set following the Shapley value formalism. Thus, kernel SHAP approximates feature contributions as Shapley values while the original LIME approach defines locality for an instance to be explained heuristically. Since kernel SHAP approximates Eq. 1, it is subject to sampling variability. Kernel SHAP requires a background data set for training. Feature absence is simulated by substituting feature values with prevalent values of training data. Then, a weighted linear regression model is trained as an explanation model *g* on the basis of artificial samples generated by setting features on or off, which corresponds to considering different feature sets. The coefficients from the model *g* are the SHAP values determining feature importance.

#### Model-dependent SHAP: decision trees

For decision tree-based models, an algorithm for the exact calculation of SHAP values has recently been reported [40]. Herein, this algorithm is adapted for compound activity and potency predictions.

## Results and discussion

Model interpretability generally depends on estimating the contribution of individual features (independent variables) to predictions. Complex non-linear models hinder interpretation but are often used in activity prediction and QSAR analysis. Accordingly, agnostic methods for consistent estimation of feature importance regardless of model complexity are highly desired. To these ends, the SHAP methodology was introduced and proof-of-concept was established by analyzing class label predictions of active vs. inactive compounds using ML approaches of different complexity including RF, SVM, and DNN [19]. Herein, we evaluate a recent methodological variant for exact calculation of Shapley values using tree-based methods and present new applications of the SHAP approach including interpretation of DNN models for the generation of multi-target activity profiles of compounds and regression models for potency prediction.

### Comparison of kernel and tree SHAP

Although model-independent kernel SHAP is generally applicable to ML models, it only approximates the theoretically optimal solution. By contrast, the tree SHAP approach yields Shapley values according to Eq. 1 having no variability. The algorithm computes exact SHAP local explanations in polynomial instead of exponential time [40].

The tree SHAP approach was applied herein to rationalize predictions of compound potency values and multi-target activity. Initially, the kernel and tree SHAP variants were systematically compared to evaluate the accuracy level of local kernel SHAP approximations in the context of activity prediction. Since the calculation of exact SHAP values is currently only available for tree-based models, two ensemble methods based upon decision trees were considered for comparison including RFs and ExtraTrees. First, global performance of RF and ExtraTree models was assessed for the 10 activity classes. Table 2 reports average model performance across these classes using different metrics (as defined in the “Materials and methods” section). Classification models for binary activity predictions and regression models for potency value predictions reached overall high performance levels and displayed low variability for different training sets.

**Table 2:** Performance of tree-based models

Method	Classification			Regression		
	AUC	MCC	BA	MAE	MSE	R^2^
RF	0.996 (0.006)	0.949 (0.048)	0.961 (0.004)	0.577 (0.077)	0.587 (0.142)	0.787 (0.073)
ExtraTrees	0.996 (0.006)	0.957 (0.041)	0.967 (0.030)	0.560 (0.073)	0.566 (0.138)	0.792 (0.072)

Reported is the mean performance (standard deviation) over 10 activity classes for decision tree-based classification and regression models using different metrics. For classification models, area under the ROC curve (AUC), balanced accuracy (BA), and Matthew’s correlation coefficient (MCC) values are given. For regression models, the mean absolute error (MAE), mean squared error (MSE), and coefficient of determination (R^2^) are reported

**Fig. 1:**
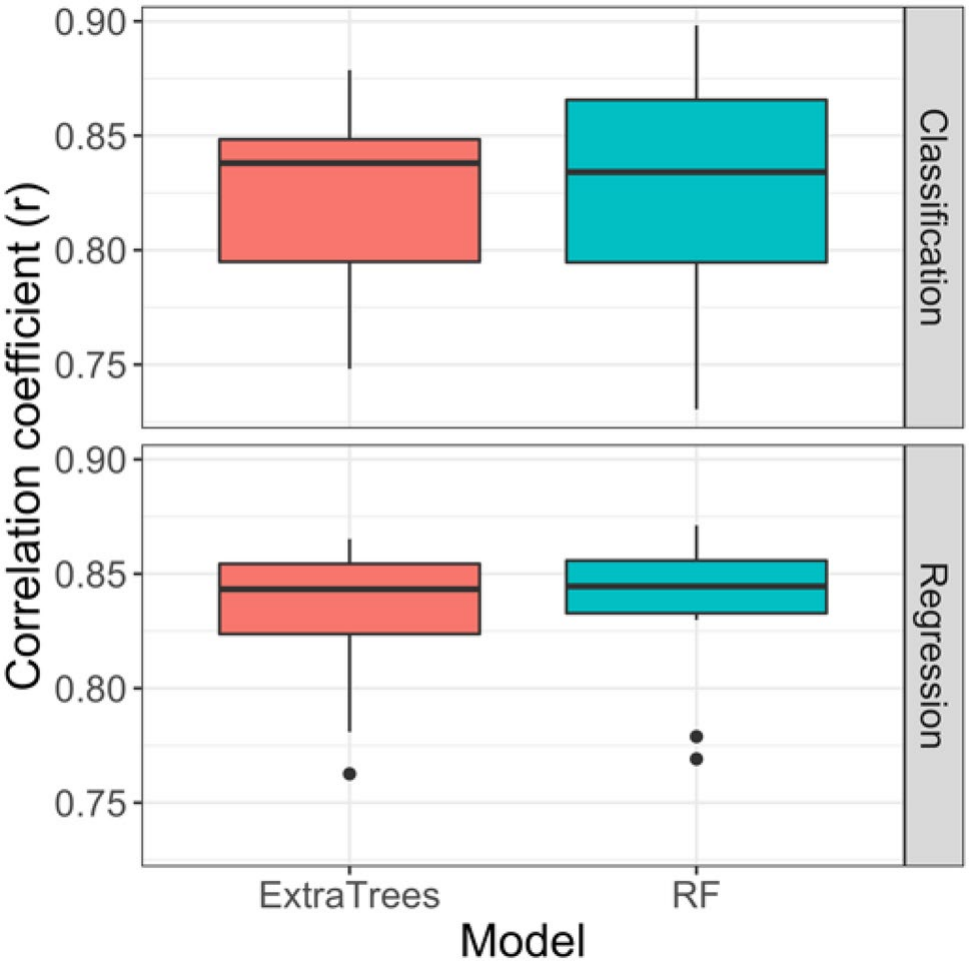
Comparison of kernel and tree SHAP. For 10 activity classes, distributions of correlation coefficient (r) values for kernel and tree SHAP calculations, corresponding to approximated and exact SHAP values, respectively, are reported in boxplots. Black horizontal lines indicate median values. Results are shown for classification (activity prediction, top) and regression (potency value prediction, bottom) models using RF (blue) and ExtraTrees (red)

Given their high performance, these models provided a sound basis for comparing the kernel and tree SHAP approaches. Figure 1 shows the distribution of correlation coefficients calculated for absolute kernel and tree SHAP values across the 10 activity classes. For classification (regression) models, the mean correlation coefficient values were 0.83 (0.82) and 0.84 (0.83) for RFs and ExtraTrees, respectively. Thus, high correlation between approximated and exactly determined importance values was observed for both classification and regression models.

A feature importance ranking was also generated for the kernel and tree SHAP approaches. For different numbers of highly ranked features, the median number of features shared by the two SHAP variants was determined. For the 10 activity classes, median values were obtained for 40 comparisons using RFs and ExtraTrees for classification and regression. Table 3 reports the number of common features for varying numbers of highly ranked features, revealing a consistently high degree of feature overlap.

**Table 3:** Top-ranked features common to kernel and tree SHAP

# Top-ranked features	Common features (Median)
5	4
10	9
20	19
30	28
40	36
50	45

Reported is the median number of features shared by kernel and tree SHAP rankings at varying numbers (#) of top-ranked features. The median value was obtained for 40 comparisons, resulting from combinations of 10 activity classes, two approaches (RF and ExtraTrees), and two prediction tasks (classification and regression)

As an additional control, the variability of kernel SHAP results was further assessed by calculating the correlation coefficient for kernel and tree SHAP for three activity classes over five independent trials. For kernel SHAP, these trials involved distinct random seeds, which influenced the generation of artificial samples for local approximations. Thus, while tree SHAP did not display variability across these trials, the use of different background data sets in kernel SHAP might influence the results. The comparison was carried out for classification models. The mean correlation coefficients for RFs and ExtraTrees were 0.73 or greater, with very low standard deviations ranging from 0.002 to 0.009.

Taken together, the findings in Fig. 1 and Table 3 indicated the reliability of local approximations from kernel SHAP, compared to exact tree SHAP solutions, and hence the utility of the generally applicable model-independent approach for the activity and potency prediction tasks investigated herein.

### Compound potency prediction

The model-dependent exact SHAP variant was then applied to explain the output values of regression models using tree-based algorithms.

#### Interpretation of gradient boosting regression

**Fig. 2:**
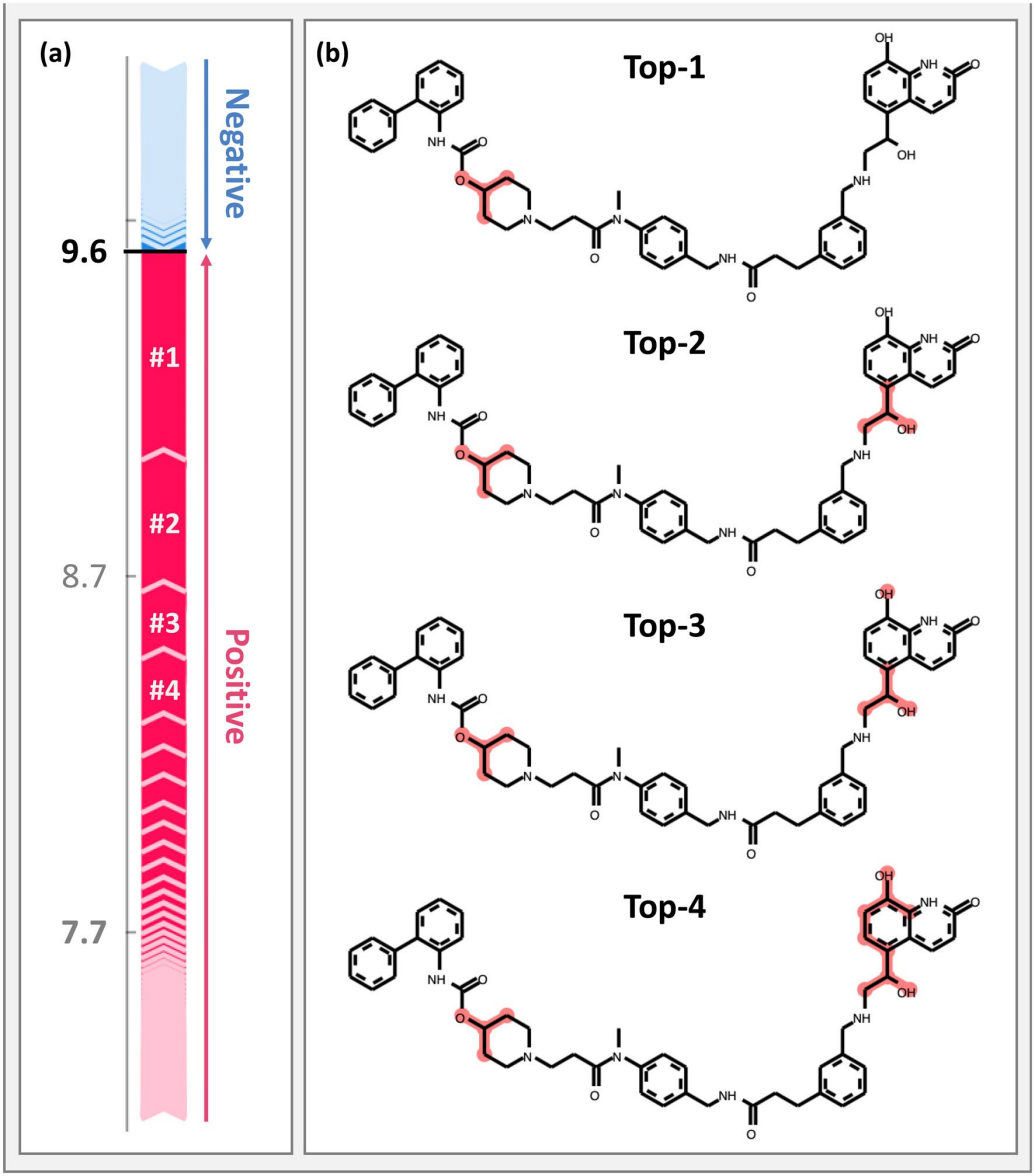
Interpretation of GB-based compound potency prediction. **a** For an exemplary prediction, a feature importance ranking is shown including features with positive (red) and negative (blue) contributions to the prediction of the high potency value. Sequential arrows on the left are proportional to the feature contributions or SHAP values (shown on the pK_i_ scale). The summation of the expected value (7.7, gray) and all feature contributions yield the predicted pK_i_ value (9.6). Numbers in white preceded by # indicate top-ranked features. **b** From the top to the bottom, top-ranked features with positive contributions are iteratively mapped onto the test compound

A GB regression model was trained to predict compound potency values of muscarinic acetylcholine receptor M3 ligands (CHEMBL ID: 245). This model predicted pK_i_ values for test compounds with MAE, MSE, and R^2^ values of 0.53, 0.52, and 0.73, respectively, and thus yielded promising results. The tree SHAP analysis framework enabled rationalizing these predictions. Figure 2 shows an exemplary SHAP-based explanation for the prediction of a compound with a pK_i_ of 10.0. This compound was the third most potent compound in the test set and was predicted by the model with an error of less than one pK_i_ unit. Figure 2a illustrates the SHAP feature ranking including positive and negative contributions. Each arrow corresponds to a given feature and its length is proportional to the estimated feature importance, i.e., the SHAP value. The expected value corresponds to the average of pK_i_ values across the training set. The sum of all SHAP values and the expected value (7.7) represents the pK_i_ value predicted by the model. This visualization indicates if there are individual features with large contributions such as the four top-ranked features (#1 to #4).  Figure 2b shows the iterative mapping of these features having the largest SHAP values on the test compound that strongly contributed to the prediction of high compound potency. As can be seen, these features defined substructures of the test compound.

To confirm that the model was indeed relying on prioritized features, systematic addition and removal of features was investigated. For feature addition and elimination, a zero-vector and the original compound fingerprint were considered as initial vectors, respectively. Then, features were added and removed randomly or according to the SHAP importance ranking. As a control for SHAP-based feature contributions, random selection of features was carried out by considering all features (*random all*), or only present features (*random present*), i.e., bits that were set on. After removal of five features the predicted pK_i_ value decreased by 2.23, 0.04, and 0.18 for SHAP, *random all*, and *random present* rankings, respectively. For random removal, reported values correspond to the average across 500 independent trials. Moreover, the addition of five individual features led to an increase in the predicted pK_i_ value of 1.72, 0.01, and 0.16 units for SHAP, *random all*, and *random present* rankings, respectively. Hence, in contrast to random selections, features prioritized by SHAP made large contributions to the prediction of high potency.

#### Interpretation of random forest regression

**Fig. 3:**
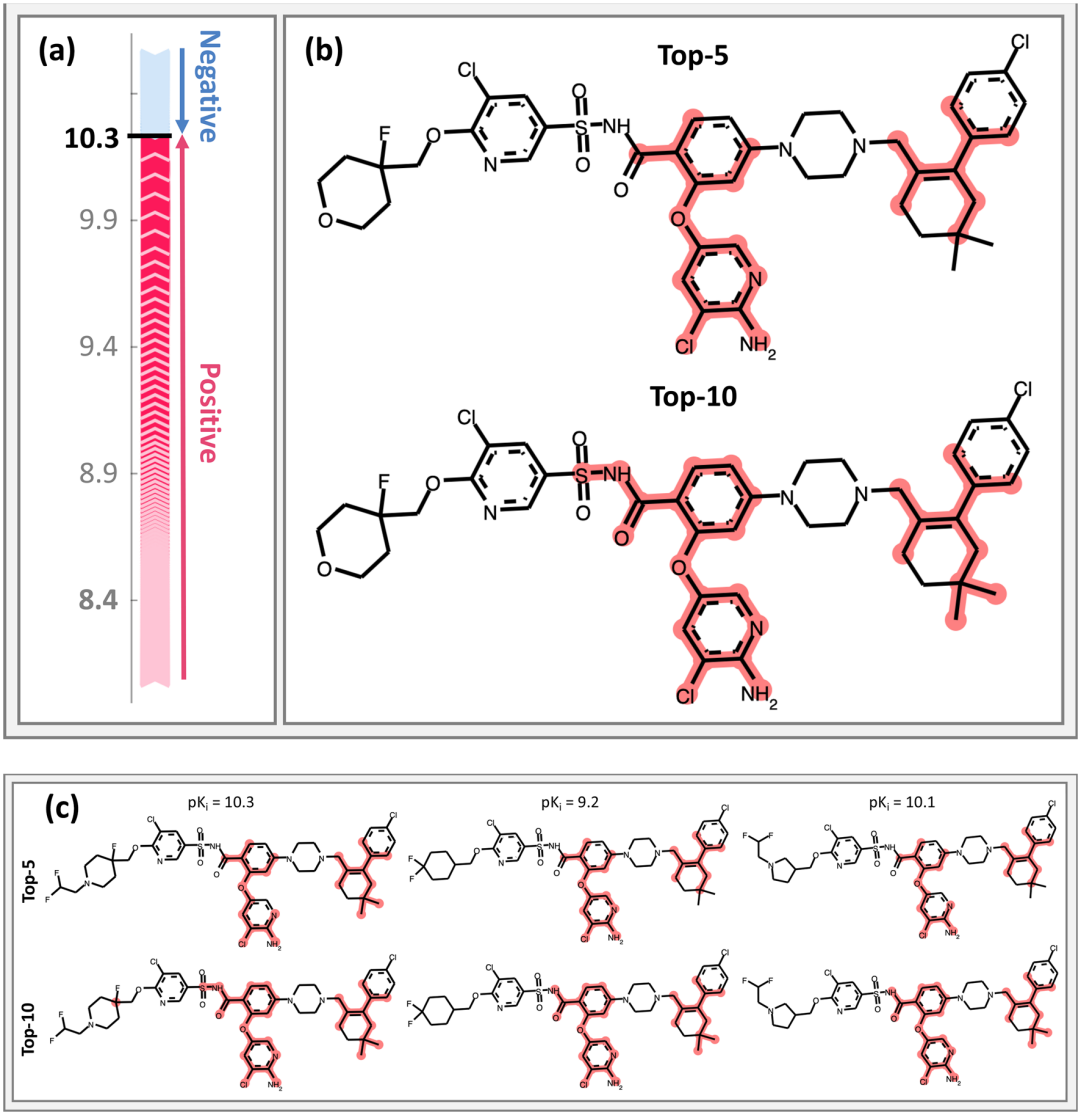
Interpretation of RF-based compound potency prediction. **a** Feature contributions to an exemplary prediction are depicted according to Fig. 2. The expected value (8.4, gray) and all feature contributions yield the predicted pK_i_ value (10.3). **b** Top-ranked features with positive contributions are mapped onto the test compound. **c** Top-5 and -10 ranked features are mapped onto three analog from the same series

Predictions from RF regression models were also interpreted applying the tree SHAP approach. The potency of apoptosis regulator Bcl-2 inhibitors (CHEMBL ID: 4860) was predicted by RF with MAE, MSE, and R^2^ values of 0.57, 0.57, and 0.78, respectively. Figure 3 shows the SHAP analysis for an exemplary inhibitor. This test compound was a highly potent inhibitor of Bcl-2, with a pK_i_ of 10.7. For this compound, the RF regression model predicted a nearly accurate pK_i_ of 10.3. Figure 3a illustrates the presence of positive and negative feature contributions. The expected pK_i_ value was 8.4 and the summation of all SHAP values yielded the output prediction of the RF model. Figure 3a shows that in this case, compared to the example in Fig. 2, many features contributed positively to the accurate potency prediction and more features were required to rationalize the prediction, as shown in Fig. 3b. Hence, SHAP analysis revealed intrinsic differences in model anatomy for comparably accurate predictions. In order to compare feature importance in closely related molecules, SHAP analysis was also applied to compounds from the same analog series (structural analogs). Three analogs from the same series were present in the test set.  Figure 3c shows the top-ranked features from SHAP analysis for these compounds. Similar features were consistently prioritized. The five and 10 most relevant features (i.e., with largest SHAP values) corresponded to very similar structural patterns for all analogs. This indicates the consistency of ranking of individual features in structurally analogous compounds. This RF regression model was trained to predict compounds from other analog series, i.e. structurally different compounds from the training set. By contrast, local models are typically trained on given analog series or structurally homogeneous data sets. In this case, the regression model prioritized corresponding structural features in analogs from the same series.

We re-emphasize that SHAP-based feature prioritization identifies features that determine ML predictions. Features that are decisive for predictions may or may not be responsible for specific activities. While correspondence between features that determine predictions and biological activities or SARs is frequently observed, there is no guarantee that features determining predictions are indeed activity-relevant. However, in the case of the Bcl-2 inhibitors shown in Fig. 3c, substructures delineated by top-ranked features such as 2-amino-3-chloro-pyridine moiety or the sulfonamide are indeed of critical importance for activity.

#### Comparison of tree-based methods

**Fig. 4:**
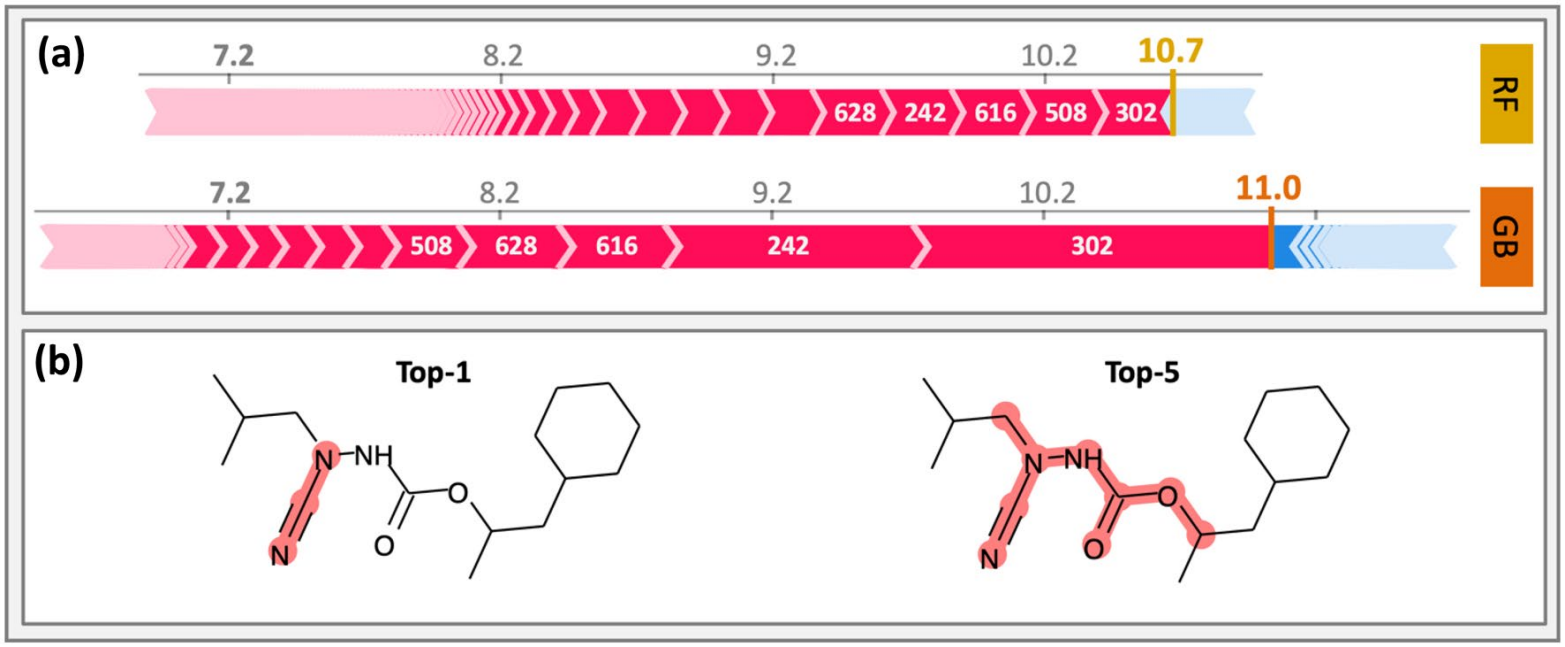
Comparative interpretation of RF- and GBM-based potency prediction. In **a**, positive (red) and negative (blue) feature contributions are compared for RF- and GBM-based regression models. The predicted pK_i_ values are shown in bold and different colors for RF (yellow) and GBM (orange). White numbers give indices of top-ranked features. **b** The top-1 and top-5 ranked features are mapped onto the compound. These features are common to both models

SHAP results were also compared for parallel application of two tree ensemble algorithms. For cathepsin K inhibitors (CHEMBL ID: 268), RF and GB regression models were generated. For RF (GB), compound potency was predicted with MAE, MSE, and R^2^ values of 0.56 (0.57), 0.55 (0.58), and 0.71 (0.70). The most potent compound in the test set had an experimental pK_i_ of 11.4. For this compound, the RF and GB models predicted pK_i_ values of 10.7 and 11.0, respectively, hence yielding another accurate prediction for a highly potent compound. Figure 4 compares the interpretation of RF- and GB-based predictions. As shown in Fig. 4a, both models included many features with positive contributions to the prediction and only few with negative contributions. In both cases, the same top-5 features were identified, albeit with varying model-dependent importance values. Figure 4b shows the mapping of the top-1 feature (index 302) and top-5 features onto the test compound. These features delineated a coherent substructure in the test compound. Feature elimination also confirmed the strong positive influence of features prioritized by SHAP on the prediction of high compound potency.

### Multi-target activity prediction

**Fig. 5:**
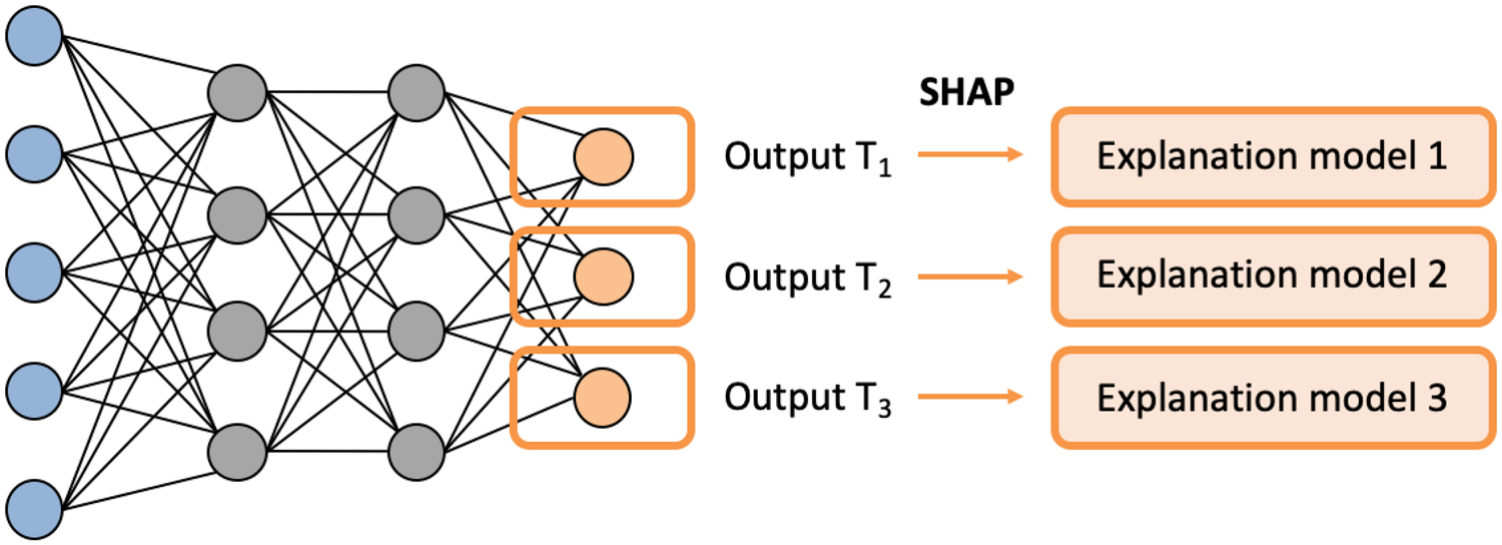
SHAP-based interpretation of MT-DNN predictions. Each output neuron facilitates activity prediction of a different target (T_x_). A SHAP-based explanation model is generated for each node/target. For a given test compound, each output prediction is rationalized

As a methodologically distinct application, MT-DNNs were trained for predicting highly and weakly potent inhibitors of different kinases and predictions were interpreted. The feasibility of such predictions was demonstrated previously [41]. The architectures of MT-DNN models contained multiple output neurons, each of which represented a different prediction task (target). Accordingly, models were derived to account for all 103 human kinases for which inhibitors were available. Each output neuron provided a binary classification output. Rationalizing predictions of multi-kinase activity of inhibitors was of special interest. MT-DNN predictions were interpretable using the model-independent kernel SHAP approach. To interpret predictions for individual targets, kernel SHAP calculations were carried out for each output neuron of the MT-DNN, as illustrated in Fig. 5. Then, multiple SHAP visualizations were combined for the comparative interpretation of activity predictions against different kinases. In the following, exemplary predictions of highly potent inhibitors of multiple kinases are discussed and model errors analyzed.

**Fig. 6:**
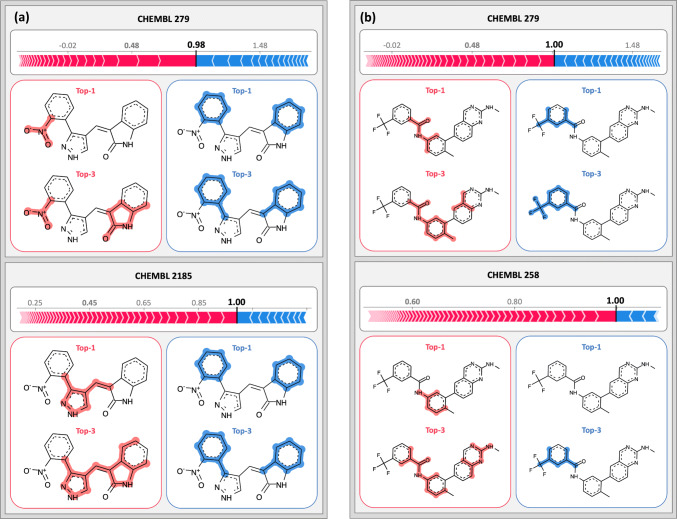
Interpretation of MT-DNN activity predictions. In **a** and **b** SHAP analysis is shown for two inhibitors that were highly potent against two kinases. The top-1 and top-3 positive and negative features are mapped onto the compound and colored according to their contributions

Figure 6 rationalizes predictions for exemplary multi-kinase inhibitors. Figure 6a shows a test compound that formed six highly potent and five weakly potent interactions with different kinases. The figure compares the SHAP analysis for two of these targets including vascular endothelial growth factor receptor 2 kinase (CHEMBL target ID: 279) and serine/threonine Aurora-B (ID: 2185) kinase. The compound was correctly predicted to be highly potent against both targets with probabilities of 0.98 and 1.00, respectively. Interestingly, features that negatively contributed to the predictions defined the same substructure for both kinases whereas features that mostly determined the correct prediction of high potency against these targets corresponded to only partly overlapping superstructures. For these kinases, the training sets differed and the models emphasized different structural features for classification as a highly potent inhibitor. Figure 6b shows the corresponding analysis for another exemplary compound with high potency against vascular endothelial growth factor receptor 2 kinase (ID: 279) and tyrosine protein kinase LCK (ID: 258). Here, features that positively or negatively influenced the predictions also delineated different substructures in these compounds. We note that the top-1 negative feature is not highlighted on the compound. In this case, the absence of a structural feature characteristic of highly potent training compounds had a negative effect on the prediction, hence decreasing the probability of activity. In fact, two of three features that contributed negatively corresponded to atom environments that were absent in the test compound (SMILES [CH]:C(:[CH])NC(:N):N and C:C([NH]):N:C:N). By contrast, all features with positive contributions were present in the compound and are mapped and highlighted in the figure. The potentially critical role of feature absence for model decisions revealed by SHAP analysis is further analyzed below.

### Insights into model errors

**Fig. 7:**
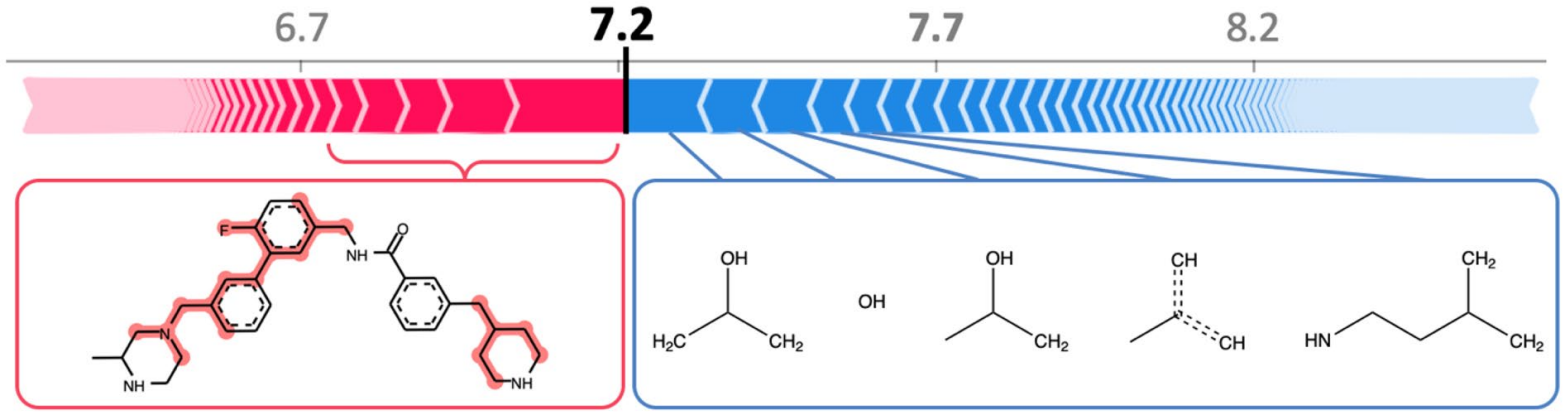
Rationalizing an error of a GB regression model. For a compound with high potency against muscarinic acetylcholine receptor M3, the potency value was ~ 1000-fold under-predicted by the GB model. SHAP identifies features making strong positive contributions to the prediction that are mapped onto the compound (left). By contrast, features with strongest negative contributions to potency prediction are absent in the compound. The corresponding atom environments are shown on the right

SHAP analysis also helps to better understand model errors and reveals reasons for inaccurate predictions. For example, the same GB regression model that correctly predicted the highly potent compound in Fig. 2 actually failed to predict the potency value of the most potent compound in the test set. Its pK_i_ value was 10.5 but the GB model underestimated its potency by 3.3 units. In Fig. 7 this compound is shown and an explanation for the model error is provided. SHAP analysis identified features making strong positive or negative contributions. The top-5 positive and negative features were mapped. In contrast to positive features, four of the top-5 negative features were not detected in the test compound. Thus, their absence made negative contributions to potency prediction. These features were prevalent in highly potent training compounds and their absence in the test compound was heavily penalized by the GB model. Had these features been present, their contributions would have been strongly positive, essentially leading to correct prediction of the high potency value. Without SHAP analysis, this type of error could not be rationalized.

**Fig. 8:**
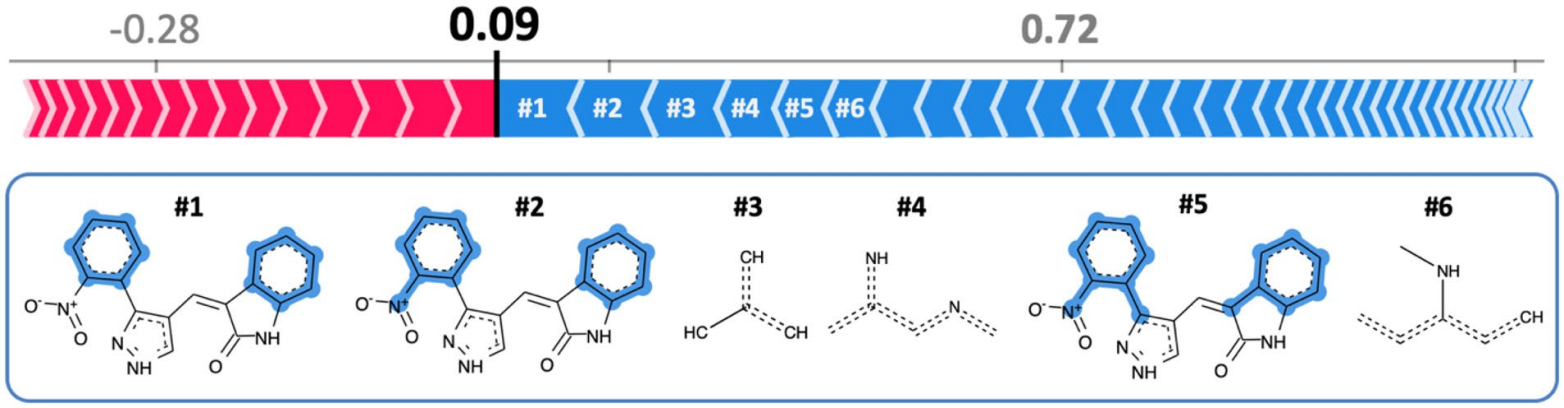
Rationalizing an MT-DNN classification error. A highly potent inhibitor of ribosomal protein S6 kinase 1 was incorrectly predicted to be weakly potent against this target. SHAP analysis identifies a variety of features with strong negative contributions to the prediction. The top-6 ranked negative features are mapped onto the inhibitor. Three of these features are present in the compound, but the three others are absent. The corresponding atom environments are displayed

Furthermore, an incorrect classification of a MT-DNN model was analyzed. In Fig. 6a, two correct predictions of high potency against different kinases were interpreted for an inhibitor. This compound was also highly potent against ribosomal protein S6 kinase 1 (ID: 4501). In this case, however, the MT-DNN model failed and predicted a weakly potent inhibitor, with a cumulative probability of only 0.09. As shown in Fig. 8, SHAP analysis identified many features that negatively contributed to this prediction, including features that were present in the inhibitor (and defined substructures) and others that were absent. Hence, in this case, multiple features with negative contributions were present in the test compound while features learned by the model to make positive contributions were absent, thus rationalizing the incorrect prediction. In these examples, the absence of features that were frequently detected in highly potent training was responsible for prediction errors. These findings illustrate the complexity of ML model decisions and also emphasize that learned features driving predictions are often model-specific.

## Conclusion

The SHAP methodology enables the interpretation of ML models and their predictions, yielding feature importance values for individual predictions from any ML model. Hence, SHAP analysis sheds light on the black box nature of many ML approaches. Once numerical values indicating the magnitude and direction of feature contributions to predictions have been determined, features can be mapped on test compounds providing intuitive visualizations of feature contributions. The kernel SHAP method was originally introduced for evaluating binary classification models. It utilizes local approximations that enable the application of the approach to ML models of any complexity including deep learning architectures; a unique characteristic of SHAP. For models based on DT ensembles, the recently developed tree SHAP algorithm makes it possible to calculate exact Shapley values, which represents the most critical step for the derivation of an explanation model. Therefore, we have been interested in further investigating the SHAP methodology, with two major goals. First, our kernel SHAP method for the assessment of compound activity prediction was compared in detail to the tree SHAP algorithm using DT-based ensemble classifiers defining its applicability domain. Local approximations we implemented make SHAP analysis generally applicable to ML models and comparing the local approach with tree SHAP made it possible to determine the accuracy level of local approximations. Second, advanced applications for SHAP were investigated including the interpretation of compound potency prediction using ML regression models and multi-target predictions using MT-DNNs, which represent complex ML scenarios. In direct comparisons, kernel and tree SHAP analysis were found to yield very similar results in the assessment of activity and potency predictions, with high correlation between prioritized features. These findings provided substantial support for the validity of the generally applicable kernel SHAP approach. Furthermore, we found that SHAP analysis yielded meaningful explanations of compound potency and multi-target predictions, revealing different model characteristics responsible for individual predictions and reasons for success or failure of a given model. For practical ML applications in drug discovery, such insights are of critical relevance. Taken together, the results of our analysis encourage further applications of the SHAP approach to better understand ML efforts and improve model quality.
